# Efficiency of Wastewater Treatment Plants (WWTPs) for Microplastic Removal: A Systematic Review

**DOI:** 10.3390/ijerph17218014

**Published:** 2020-10-30

**Authors:** Antonio Cristaldi, Maria Fiore, Pietro Zuccarello, Gea Oliveri Conti, Alfina Grasso, Ilenia Nicolosi, Chiara Copat, Margherita Ferrante

**Affiliations:** Department of Medical Sciences, Surgical and Advanced Technologies “G.F. Ingrassia”, University of Catania, Via Santa Sofia 87, 95123 Catania, Italy; antonio.cristaldi81@gmail.com (A.C.); mfiore@unict.it (M.F.); pietro.zuccarello@unict.it (P.Z.); alfina_@hotmail.it (A.G.); ileniatiziananicolosi@yahoo.it (I.N.); ccopat@unict.it (C.C.); marfer@unict.it (M.F.)

**Keywords:** microplastics, wastewater, wastewater treatment plants, removal efficiency, water contamination, PRISMA

## Abstract

Plastic is widely used for human activities (food packaging, medical, technological devices, etc.) and there is a growing concern regarding the risks for environmental and human health because they have still not been fully evaluated. Particularly, microplastics (primary and secondary) are present in all environmental compartments and this poses a potential threat because of their entry into the food chain. Furthermore, microplastics can absorb numerous pollutants that can be accumulated in the human body through bioaccumulation and biomagnification processes. We carried out a systematic review using a PRISMA approach to verify the efficiency of wastewater treatment plants (WWTPs) for microplastic removal. The international databases (PubMed, Science Direct, Scopus) were used to find published studies on efficiency of wastewater treatment plants (WWTPs) for microplastic removal. The search period was between January 2010 and June 2020. Over 1000 full research papers were initially selected through the use of keywords. After that, the papers were further selected by English language, title, and abstract, and duplicate papers and non-relevant papers were eliminated according to eligibility criteria. Finally, we included 15 full research papers. In each of the 15 full research papers selected, the microplastics identified were categorized by the authors for shape, size, and type of polymers identified. The characterization of the various types of microplastics was performed by Fourier Transform Infrared Spectroscopy (FTIR) or Raman spectroscopy. We have observed how wastewater treatments plants located in different continents (Europe, Asia, North America) mostly use a primary and secondary type of treatment that allows one to reach a high percentage of microplastics removal from wastewater. Most of the wastewater treatments plants investigated reported a microplastics removal efficiency greater than 90%, but despite this, millions of microplastics continue to be released every day into the aquatic environment. Then, in the near future, efficient and common standardized protocols for monitoring MPs should be drawn up, as well as increasing the knowledge of sources and strategies to further reduce microplastics contamination of treated wastewater.

## 1. Introduction

Over the years, the growing use and incorrect recycling of plastic have significantly increased the presence of this contaminant in all environmental matrices. Agricultural activities (e.g., use of fertilizers, greenhouse covers, etc.), illegal dumping of waste, and atmospheric deposition have a significant impact on soil, while fishing related activities, the illegal dumping of plastic waste, and wastewater treatment plants (WWTPs) have a significant impact on water. Several studies showed that wastewater treatment plants are a source of release of microplastics (MPs) into the aquatic and terrestrial environment, including after treatment [1,2,3]. So, through the sewage system, millions of microplastics pass through WWTP and the characteristics of microplastics depend on local agricultural, urban, and/or industrial activities. All microplastics that are not retained by WWTP will reach rivers and consequently, the oceans [2,4,5,6,7].

Furthermore, the concentration of microplastics is also influenced by seasonality, given that it is higher in hot periods because exposure to the sun favours the fragmentation and degradation of plastic. Rain also has a significant impact due to the entry into the sewage system of microplastics washed out of the ground. However, no differences were found for the microplastics concentration between the day and night [2,8].

Plastic is now used for the production of many everyday products and given its characteristics, it seems difficult to do without it. It has been reported that over 330 million tons of plastic were produced worldwide in 2016 [1]. If on the one hand plastic is used for various human activities (food packaging, medical, technological devices, etc.), on the other, there is a growing concern regarding the risks for environmental and human health because these still have not been fully evaluated [9,10].

EFSA (European Food Safety Authority) has defined microplastics as synthetic polymeric compounds with a diameter of less than 5 mm and nanoplastics as those with a diameter of less than 0.1 μm [1,11]. Furthermore, the microplastics have been classified as primary and secondary based on their origin [1,2]:

- primary microplastics derive from personal hygiene products, cosmetics, detergents, and fibers released from laundry;
- secondary microplastics are originated as a result of plastic fragmentation, which is released into the environment after chemical-physical and/or biological interactions.

As a result, microplastics (primary and secondary) are present in all environmental compartments and this poses a potential threat, not only to animals, but also to humans, because of the entry of microplastics into the food chain [2]. In addition to the ingestion of microplastics, the inhalation of microplastics present in the air and dermal contact should also not be underestimated [2].

Microplastics can absorb numerous organic pollutants such as DDT, polychlorinated biphenyls, dioxins, PAHs, but also trace element like heavy metals [12,13,14,15] and other harmful agents such as pharmaceuticals and pathogenic organisms [1,2,7]. Therefore, microplastics act as a vector for these pollutants that can be accumulated in the human body through bioaccumulation and biomagnification processes [1,16]. Once entered into the human body, even at very low concentrations, they can cause many health problems like the risk of contracting cancer or reproductive disorders, immune system disorders, endocrine dysregulation, and an increase in birth defects [1,9].

WWTPs have a high efficiency of microplastics removal (according to the type of treatment, primary, secondary, or tertiary), but they still represent a critical point of contamination because water remains the main carrier for the spread of microplastics [1,2,3,5,8]. In general, primary and secondary treatment are those that have the greatest ability to remove microplastics, with values ranging from 78% to 98% and from 7% to 20%, respectively [2,16]. Tertiary treatment, on the other hand, does not seem to have significant effects on reducing the concentration of microplastics. As for pretreatment processes, Murphy et al. [17] report that maximum microplastic removal efficiency can be achieved during the grit and grease removal process [2,7,17]).

The typology, shape, size, and quantity of the microplastics present in the WWTP effluent are variable and all this makes it difficult to compare the various plants present in different countries because other factors such as methodological differences also come into play [1,3]. The microplastics that are found in the samples taken in the wastewater treatment plants can be classified into six main shapes: fibers, granules, pellets, films, foams, and fragments. Usually, fibers and fragments are the most frequent shapes and the fibers are more difficult to remove due to their high length to width ratio [2].

From the data in the literature it is now clear that the amount of microplastics released by WWTPs all over the world is worrying, given that millions of particles are released every day in the environment [1,3].

Microplastics found in domestic wastewater can originate from different sources, such as personal care products and cosmetics (PCCP): for example, exfoliating creams, soaps, toothpaste, shampoo, etc., represent an important source of microplastics. The microplastics present in these products are mostly made of polyethylene and are called microbeads or microsphere. However, it should be highlighted that the name does not exactly reflect the geometry of the plastic particles present in the PCCP because it is a material that almost always shows an irregular shape and, in general, it has dimensions less than 1 mm [1,18,19]. The microspheres can be present in cosmetics for a percentage that varies between 0.5% and 5%, with an average size of 250 μm. In toothpaste, microspheres are used because they remove plaque and stains thanks to their abrasive action [2,20].

About 35% of the microplastics found in the oceans derive from the washing processes of synthetic fiber fabrics [2,3]. Textile fibers, which represent about 70% of the microplastics from WWTPs, are also commonly found in wastewater. Some studies showed that a laundry can release more than 1900 polyester fibers (polyester terephthalate) at each wash and this number may increase according to the type of fabric: for example, a fleece garment could release about 110,000 fibers and 5 Kg of polyester fabrics up to 6,000,000 microfibres [1,21,22,23,24]. The amount of fibers released depends on the type of fabric, but also on other factors such as temperature, time, washing speed, and the type of detergents and fabric softeners used. Therefore, fibers and microfibres will be released by the WWTP into the environmental matrices and their presence will not be due only to water contamination, but also to sludge generated by WWTP [1,22,23,25].

Other personal use products that can release microplastics into domestic wastewater include glitter, contact lenses, and jewelry. In addition to these, there are also non domestic sources that release microplastics into wastewater, including: (a) plastic particles used in airblasting processes; (b) plastic resin pellets during production and/or transport processes; (c) lost styrofoam used in fillings or shipping; (d) synthetic fibers released by textile industries; (e) dust from drilling and cutting plastics. Due to their release in drains or sewage systems, it is therefore necessary to identify and quantify the contribution of these sources, considering all the products and types of polymers, so as to develop adequate methods to reduce their release [7].

Despite the treatment of wastewater, the treatment plants themselves constitute a source of microplastics in the aquatic environment and through the sludge, in the soil. All this, therefore, represents an important problem for the health of our planet and consequently, for humans.

With this review, we want to provide an updated state of the art review of the last decade (2010–2020) on the efficiency of WWTPs for the treatment and removal, identification, and shape of microplastics, as well as their eventual release and fate in the environment.

## 2. Material and Methods

Research was performed according to the PRISMA criteria [26]. The international databases (PubMed, Science Direct, Scopus) were used to find published studies. We filtered only full research articles published in English language and selected the following keywords: microplastics in wastewater, microplastics in WWTPs, microplastics removal by WWTPs, microplastics pollution after WWTP.

The search period was between January 2010 and June 2020 and only full text research papers were included. Letter, opinion, commentary, review, or non-relevant articles were excluded. Additional eligibility criteria were:

- use of glass bottles for sampling;
- steel sieves;
- glass fiber filters;
- any other laboratory materials in glass;
- use of cotton coats by operators to prevent contamination of fibers;
- preparation of control samples to determine the possible level of contamination by microplastics.

The papers selected in this way allowed us to collect data based on:

- type of treatment and origin of the water treated by WWTPs (domestic, municipal, and industrial);
- size, shape, and type of microplastics treated in WWTPs;
- microscopic technique used for the characterization of the different types of microplastics.

So, the papers selected as suitable have provided indications on the removal potential and release of microplastics from WWTPs, but also the techniques for their separation and identification, as well as the current state of knowledge and their environmental fate.

**Figure 1:**
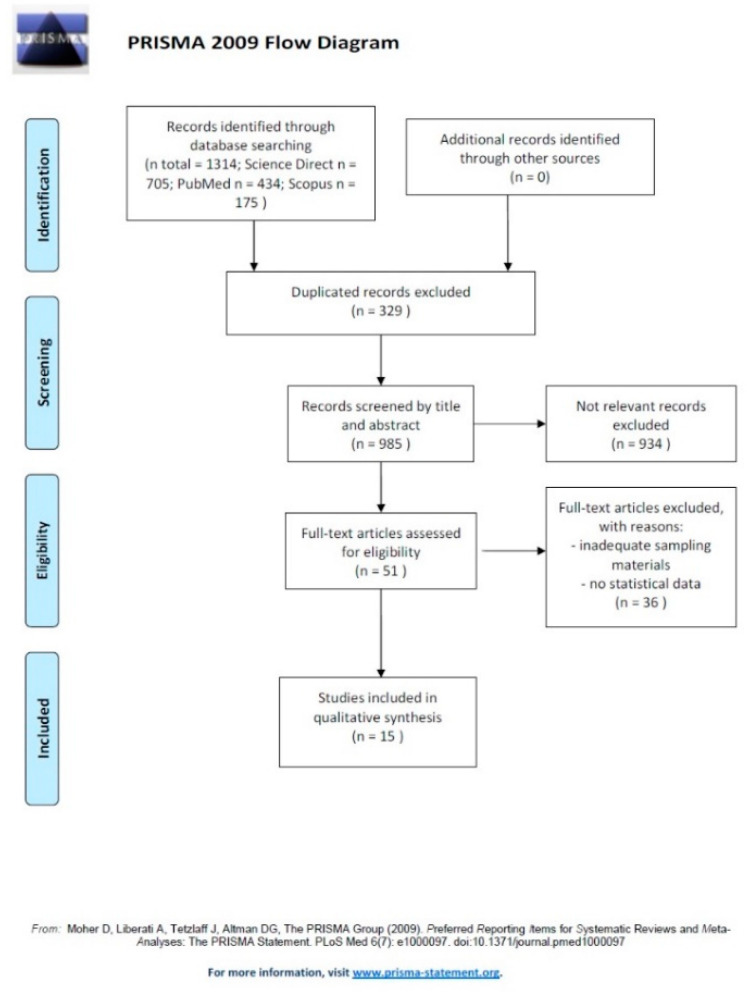
PRISMA flow diagram.

Our initial search produced 1314 potential literature references (Figure 1). We removed 329 duplicate full research articles; after an accurate review of the abstracts and titles of 985 full research articles, we screened 51 full research articles as eligible papers for the final quality evaluation. Of these, we excluded 36 for poor quality and/or insufficient data (e.g., inadequate sampling materials, no statistical data). Finally, we included 15 full research articles, in particular 4 from China, 3 from Spain, and 1 each for Canada, Denmark, Germany, Italy, Scotland, South Korea, Turkey, and USA (Table 1).

## 3. Results and Discussions

Our systematic review provided 15 full research articles [4,5,8,17,27,28,29,30,31,32,33,34,35,36,37] included in the qualitative synthesis (Table 1).

Akarsu et al. [27] carried out a study in Mersin Bay (Turkey) during 2017 to evaluate the quantity, shape, and type of microplastics in influent and effluent waters from three main municipal WWTPs (Karaduvar, Tarsus, and Silifke) and understand their role in microplastic pollution in the north-eastern Mediterranean.

The Karaduvar plant applies tertiary treatment processes, which included screening, ventilated sand and an oil chamber, preliminary sediment tank biological and chemical phosphorus removal units, aeration tanks, final sediment tank, followed by deep sea discharge. Instead, the other two treatment plants (Tarsus and Silifke) applied secondary treatment processes, which included screening, preliminary sediment, aeration tanks, and final sediment tanks.

The particles present in the water samples, divided by color, size, and shape, were detected under a microscope coupled to FTIR and were classified into four main shapes: fiber, soft plastic, hard plastic, and others (polystyrene, rubber, etc.).

MPs found in the influent and effluent waters of Tarsus and Silifke showed annual average lengths between 1057 and 1095 μm. The Karaduvar WWTP showed higher values for both influent water (average annual length 1242 μm) and effluent water (average annual length 1499 μm).

T-test analysis was used to evaluate possible differences in the particle size of microplastics from influent and effluent waters, while possible trends in monthly microplastic data were investigated using the Mann-Kendall statistical test. Contrary to what was expected, the mean length of MPs in the effluents (1309 μm) was greater than that of the influents (1135 μm). The difference in mean size between the influent and effluent values was confirmed by the *t*-test (*p* < 0.001). Mann-Kendall test showed no apparent trend in monthly microplastics data for influent (Kendall’s tau = −0.183, *p* = 0.435) or effluent samples (Kendall’s tau = +0.183, *p* = 0.435).

Over the entire sampling year, it emerged that the predominant form of particles in the three WWTPs were fibers, with a percentage of 69.7%, particularly in influent waters (79%). Instead, the percentage of hard plastics has substantially increased (from an annual average of 7.5% to 32.8%) in effluent water. The analysis carried out through FTIR spectroscopy confirmed the presence of plastic polymers in the effluent: 51% was of polyethylene (PE), 35% was of polypropylene (PP), 6% was of acrylic fiber, 4% was of polystyrene (PS), and 4% was of cellulose acetate. Further, 13 fiber particles analyzed by FTIR were composed of 6 PP, 2 PS, 3 acrylic fibers, and 2 cellulose acetate, but none were found to belong to the PE group.

Several studies [17,24] have reported that with primary treatment, an MPs removal efficiency ranging from 78% to 98% can be obtained, while the secondary treatment is responsible for a small decrease in MPs, ranging from 7% to 20%, and tertiary treatment appears to have no effect on the concentration of MPs [24,38]. As reported in the literature, it was further confirmed in the study by Akarsu et al., [27], in fact, despite the WWTP of Karaduvar being equipped with a tertiary treatment system, it showed the lowest removal efficiency (38%) compared to the Silifke (58%) and Tarsus (78%) plants, which use a secondary treatment process. Thus, in total, the three WWTPs showed a removal efficiency of 58%.

Bayo et al. [28] evaluated the microplastics removal through two technologies, MBR (membrane bioreactor) and RSF (rapid sand filtration), in a WWTP located in the region of Murcia, in the south-east of Spain. In this WWTP, both domestic and industrial wastewater were treated and it consists of a double flow plant, with an anoxic tank, a biological reactor, and a membrane filtration tank, where the MBR unit is submerged.

The use of the bioreactor membrane (MBR) has shown a high efficiency for the removal of microplastics that can potentially reach 100% (99.9%). This technology uses the combination of a membrane filtration processes with suspended growth biological reactors that allow only the smallest particles to cross the system. However, it is very expensive, particularly the membrane, and also requires high energy, control of scale, and low flow [2].

Rapid sand filtration (RSF) is one of the low cost technologies that allows rapid pollutants removal and although it has shown a removal efficiency of microplastics up to 97% (45–97%), it has the main disadvantage that it can fragment microplastics into smaller particles [2,7,39].

The monitoring lasted 18 months (February 2018–July 2019). The authors divided the microparticles by color, shape, and size and made a first visual classification as bead, a spherical piece, fragment, fiber, and film, followed by a classification by size: mini microplastics (smaller than 1 mm) and microplastics (between 1 and 5 mm). Finally, the microplastics were divided into microplastic fibers (MFBs), i.e., pieces of filament with a length of at least five times its width, and microplastic particles (MPPs).

FTIR spectroscopy has been used for the identification of functional groups and molecular composition of polymeric surfaces.

The average microplastic concentration found was 4.40 ± 1.01 MPs L^−1^ for the influent, 0.92 ± 0.21 MPs L^−1^ for MBR, and 1.08 ± 0.28 MPs L^−1^ for RSF, without statistically significant differences for MPs removal between MBR and RSF (F-test = 0.195, *p* = 0.661). The main types of MPs found were fibers (1.34 ± 0.23 items L^−1^), films (0.59 ± 0.24 items L^−1^), fragments (0.20 ± 0.09 items L^−1^), and beads (0.02 ± 0.01 items L^−1^).

Fourteen types of polymers have been identified in the influent. Persistent low density polyethylene (LDPE), nylon (NYL), and polyvinyl (PV) were identified in the RSF effluent, while only melamine (MUF) after MBR treatment. In general, LDPE was the most present polymer (70.61%), followed by high-density polyethylene (HDPE) (5.16%), acrylate (AC) (4.97%), polypropylene (PP) (4.95%), polystyrene (PS) (4.02%), nylon (NYL) (3.01%), methacrylate (MCR) (1.67%), polyvinyl (PV) (1.63%), poly (ethylene: propylene) (EPM) (1.01%), and melamine (MUF) (1.01%). Furthermore, to a lesser extent, other polymers have also been found such as biopolymer (BPL) (0.59%), polyester (PEST) (0.53%), teflon (PTFE) (0.42%), and polyisobutylene (PIB) (0.42%).

MBR showed a total removal efficiency of 78.24% and RSF of 74.68%. The removal efficiency of the microplastic particulate (MPP) was 98.83% for MBR and 95.53% for RSF, whereas for the fibers (MFBs), the removal percentage was lower—57.65% for MBR and 53.83% for RSF.

Although the two technologies showed good efficiency in removing MPPs, the main problem continues to be represented by MFBs as they are able to bypass the MBR due to the high pressure applied in this system and are released into the aquatic environment. Furthermore, given the small size and morphology of the fibers, the RSF also allows them to pass into the aquatic environment [28].

Bayo et al. [8] reports abundance, shape, and type of microplastics (MPs) present in WWTP, located in Cartagena (Spain), taking into account water parameters and environmental factors, possible sources, and removal efficiency of microplastics.

It is a WWTP that receives both urban and industrial wastewater and it consists of an activated sludge process (ASP), where wastewater treatment takes place in four steps: grit and grease removal (GGR), primary clarifier (PCL), activated sludge reactor (BRT), and the effluent after the secondary clarifier (EFF).

The sampling was carried out from September 2016 to April 2018. A total of 1163 ML particles (microlitters, i.e., polymer particles less than 5 mm in diameter), divided by shape, size, and color, were identified on all wastewater samples by visual examination with a stereomicroscope and identification of functional groups and the molecular composition of the polymeric surfaces through FTIR spectroscopy.

The mean concentration of ML was 12.43 ± 2.70 ML L^−1^ for GGR, 9.73 ± 3.04 ML L^−1^ for PCL, 3.21 ± 0.50 ML L^−1^ for BRT, and 1.23 ± 0.15 ML L^−1^ for EEF. WWTP showed a ML efficiency removal of 90.1%. A statistically significant decrease in ML particles from PCL to BRT of 67.0% was found (*t*-test = 2.257, *p* = 0.042). The MPs comprised 46.6% of total ML particles with average concentrations of 3.20 ± 0.67 MPs L^−1^ for GGR, 2.59 ± 0.85 MPs L^−1^ for PCL, 2.13 ± 0.38 MPs L^−1^ for BRT, and 0.31 ± 0.06 MPs L^−1^ for EFF. WWTP showed a MPs efficiency removal of 90.3%. The most significant decrease (85.4%) was observed between BRT and EEF (*t*-test = 4.947, *p* = 0.000), although unlike the ML, there was no statistically significant difference between PCL and BRT (*t*-test = 0.395, *p* = 0.700). These results could indicate a clear influence of the biological reactor in the removal of MPs.

At the end of the process, this WWTP showed an MPs removal efficiency of 90.3% in the final effluent and a total of 542 MPs were identified as present in five different forms: fragments (46.9%), films (34%), beads (11.5%), fibers (7.4%), and foam (0.2%).

The mean size of the MPs was 0.82 ± 0.06 mm in GGR, 0.74 ± 0.08 mm in PCL, and 0.63 ± 0.03 mm in BRT, showing that in the main phases of wastewater treatment, there was a statistically significant removal of MPs (F-test = 3.038, *p* = 0.029). At the level of EEF (0.83 ± 0.14 mm), there was a statistically significant increase compared to BRT (F-test = 4.880, *p* = 0.028), although there was no corresponding increase in fiber concentration.

In total, 17 types of polymers were identified and the highest concentration was given by LDPE (2.83 ± 0.47 L^−1^), followed by HDPE (high-density polyethylene) (0.94 ± 0.41 L^−1^), ACRYL (acrylate) (0.83 ± 0.30 L^−1^), PP (polypropylene) (0.64 ± 0.11 L^−1^), PEP (polyethylene propylene) (0.27 ± 0.09 L^−1^), PS (polystyrene) (0.21 ± 0.10 L^−1^), BPL (biopolymer), NYL (nylon) (0.19 ± 0.07 L^−1^), PUR (polyurethane) (0.14 ± 0.08 L^−1^), PET (polyethylene terephthalate) (0.13 ± 0.06 L^−1^), MCR (methacrylate) (0.11 ± 0.07 L^−1^), PTFE (Teflon) (0.07 ± 0.06 L^−1^), MMF (melamine) (0.04 ± 0.02 L^−1^), PES (polyester), PVI (polyvinyl) (0.03 ± 0.01 L^−1^), PIB (polyisobutylene) (0.02 ± 0.01 L^−1^), and RBB (rubber) (0.01 ± 0.00 L^−1^).

Conley et al. [4] determined the removal efficiency of three WWTPs: the Charleston Water System, located in the city of Charleston, South Carolina (USA), which serves the Plum Island area, and Mount Pleasant, which operates two conventional wastewater treatment plants of activated sludge (Center Street and Rifle Range Road). The three WWTPs receive residential, commercial, and industrial waste. These WWTPs, with different treatment sizes, operations, and service compositions, discharge in Charleston Harbor, South Carolina (USA). Sampling was conducted in June and October 2016 and in January, April, and July 2017. During this period, more than 200 samples were collected and characterized.

The Plum Island plant receives the largest load of MPs per day in influent in respect to Rifle Range and Center Street. Fibers, as in other studies [40,41], represented the most type of MPs present, with an average of 75% or more in all samples. Regarding the MPs present in the effluents, the authors report that the mean concentrations of MPs at Plum Island were statistically significantly lower than for Rifle Range and Center St.

Based on their size, the microparticles were divided into three categories: 60–178 μm, 178–418 μm, and >418 μm. The concentrations of the effluents in all three WWTPs ranged from 1 to 30 MP counts/L and the differences in MPs concentration in the final effluent could be due to several factors, such as the characteristics of the WWTPs (i.e., treatment process and technology, flow rate, service population, service compositions), differences in the sampling method and processing methods (i.e., grab vs. composite sampling, microplastic isolation, and analysis methods), and finally, the sampling frequency. Plum Island showed the greatest effectiveness in removing MPs with a value between 95.9% and 98.1%, while Rifle Range and Center St. had a value ranging from 74.8% to 97.1%. It is probable that the variations in the removal efficiency among WWTPs are due to differences in the treatment units that could affect MPs concentrations in influents. In addition, the Plum Island plant has four primary clarifiers, with hydraulic holding times of approximately 2 h, which promote solid sedimentation before biological treatment takes place. In each primary clarifier, the floating solids are skimmed from the surface of the supernatant water before performing the secondary treatment. Depending on the density, MPs can be removed by flotation or sedimentation. In a flotation process, a gas (usually air) is injected into the lower part of a tank (called a “flotation cell or reactor”) where the water to be treated is collected, while an agitator homogeneously disperses the air. There will be an interaction between the hydrophobic air bubbles and the microplastics, which are also hydrophobic, and in this way, the air bubbles with the microplastics rise towards the surface, while the hydrophilic particles remain in the liquid phase. Consequently, it will be possible to collect the supernatant, containing the microplastics, in a collection container. The flotation process is performed before sedimentation in order to improve its performance. Another process that can improve the sedimentation process is elutriation. Briefly, the elutriation process allows one to separate the microplastics from the sediments. It is a methodology designed at a lab scale to separate the heaviest particles from the lightest ones based on their sedimentation rate when they are suspended in an upward stream flow (often a liquid) and depends on size, density, and particle shape. This process showed a 93–98% microplastic removal efficiency [2,16].

At the end of the treatment process, the fiber removal potential was lower than the plastic particle removal potential in all three WWTPs (% fiber removal 97.2 ± 1.0, 80.2 ± 8.0, 83.7 ± 8.2 versus % particle removal 98.4 ± 1.3, 95.4 ± 2.4, 88.8 ± 9.6 for Plum Island, Rifle Range, and Center Street, respectively), representing an important environmental problem due to their release into the aquatic environment. The total of MPs removed was 97.6 ± 1.2% for Plum Island, 85.2 ± 6.0% for Rifle Range, and 85.5 ± 9.1% for Center Street.

Only in this study, among the 15 selected, the authors did not specify the type of the different polymers found in the wastewater samples collected, but they did perform the subdivision into fibers and other plastic particles. Polymers typing can help to identify and mitigate sources, as well as be able to assess the potential toxicological damage posed by materials. However, given the importance of the data provided, we decided not to exclude this study from our review.

Edo et al. [5] studied the presence of microplastics in primary and secondary effluents and in the mixed sludge of a WWTP located near Madrid (Spain), as well as the presence of MPs in dried sludge and marketed as soil improvers. The plant consisted of a primary clarifier followed by A2O biotreatment and the effluent discharges to the Henares River, in the Tagus basin.

The sampling was carried out over three different days in three different months during the spring of 2019. A visual inspection was carried out, also taking into account the shape and color, and the microparticles were counted using a stereomicroscope. The total of the particles found included natural fibers with evidence of anthropogenic process, natural materials, and a series of unidentified fiber fragments. The identification for each type of particle was performed through FTIR spectroscopy.

The authors divided the microparticles into fragments (small particles, films, or beads) and fibers, defining the fibers as microparticles with a cylindrical shape and length/diameter ratio > 3 as reported by ECHA.

Based on the size, the microparticles were divided into three categories: 25–104 μm, 104–375 μm, and >375 μm <5 mm. The microparticles most present were those belonging to the group with the smallest dimensions: 54% (25–104 μm), 34% (104–375 μm), and 12% (>375 μm <5 mm) in primary effluent and 48%, 28%, and 23% in secondary effluent. Twelve different anthropogenic polymers and groups of polymers were identified in the wastewater sample and 51% were fragments. In the case of manufactured natural polymers, 62% was made up of fibers. Although manufactured natural polymers are not plastic materials, the authors have paid attention to these products given their similarity to plastic particles and the possible risk if released into the environment because their properties are changed following the manufacturing processes (in particular, for the presence of dyes).

The main polymers, in addition to dyed cotton, found in the primary effluent in descending order were: polyester fibers, polyethylene (PE), polypropylene (PP), and cellophane fibers; in the secondary effluent, the order was: PE, polyester fibers identified as PET (polyethylene terephthalate), PP (polypropylene), and cellophane. Polyester fibers were also more present in the sludge, followed by acrylic fibers, PE, and PP. Other polymers identified were polymethylmethacrylate (PMMA), polycaprolactone (PCL), polyurethane (PU), and polystyrene (PS).

At the end of the treatment process, the plant showed a high efficiency of 93.7% in the removal of microplastics from the wastewater. In fact, the total concentration of MPs was 171 ± 42 particles/L in the primary effluent, which was reduced to 10.7 ± 5.2 particles/L at the outlet of the secondary settler. The results obtained showed a high efficiency on the part of WWTP, but despite this, a high number of particles are released into the environment. The authors report that approximately 300 million microplastic particles are released into the Henares River daily, with an approximate microplastic load of 350 particles/m^3^.

Gies et al. [29] evaluated the efficiency of separation, cleaning, counting, and characterization of MPs in samples from a regional WWTP serving Vancouver, Canada.

This plant treats municipal wastewater and stormwater; it involves a first phase of removal of large debris, followed by a primary clarification and biological degradation and after that, a secondary clarification, chlorination (seasonal only), and discharge into the Fraser River upstream of the marine waters of the Strait of Georgia.

The wastewater sampling was carried out at the level of the influent and primary and secondary effluents on 16 September, 29 September, and 28 October 2016.

The possible MPs were counted, measured, and classified by shape (fibers, foam, granules, sheets, pellets, or fragments) and color and their identification was carried out through FTIR spectroscopy.

Taking into account the size, the MPs were divided into two categories: <500 μm, >500 μm, and the smallest particle had a size of 65 μm.

Analyzed samples showed a predominance of fibers (65.6%) and fragments (28.1%), followed by pellets (5.4%), while only a small percentage was given by foam, granules, and sheets (0.22%, 0.45%, and 0.20%, respectively). Analysis of the variance showed that the composition of the MPs in wastewater influent differed from primary and secondary effluent samples (*p* = 0.008 and *p* = 0.004, respectively), while the composition of the MPs in the primary and secondary effluent were no different from each other (*p* = 0.8).

The influent wastewater had 31.1 ± 6.7 suspected MPs/L, reduced to 2.6 ± 1.4 suspected MP/L, i.e., 91.7% after the primary clarification. The remaining fibers and suspect particles were reduced by 92.8% and 88.4% after primary sedimentation and foam removal. After the secondary treatment, a further reduction of 5.6% for the fibers and 9.5% for the particles was obtained, achieving an MPs removal efficiency of 98.3%. Finally, 32% of the suspected MPs have been confirmed as plastic polymers, mostly polyester (PEST). The others were polyamide (PA) and polystyrene (PS) for the particles, while polystyrene (PS), PA (nylon), and polypropylene (PP) for the fibers.

Although a high percentage of MPs removal has been achieved by the largest WWTP in Vancouver, also in this study, the authors underline that the main problem remains the incomplete removal of MPs as they have estimated that 30 billion MPs are released from the plant every year, leading to consequences for the biota that are still not entirely clear.

Liu et al. [30] observed the variation of MPs in the different treatment phases of a WWTP in Wuhan, China, so as to identify and quantify the MPs present in municipal and industrial wastewater that discharges in Yangtze River.

The treatment process involved four phases: inlet of coarse grid (Influent, W1), outlet of the primary sedimentation tank (W2), outlet of the secondary sedimentary tank (W3), and outlet of chlorination disinfection (Effluent, W4).

The MPs were distinguished on the basis of quantity, size, shape, and color and the identification of the type of MPs found was performed with the micro-Raman spectroscopy.

The quantification of MPs in WWTP was 79.9 ± 9.3, 47.4 ± 7.0, 34.1 ± 9.4, and 28.4 ± 7.0 n L^−1^ for W1, W2, W3, and W4, respectively, reaching a total removal rate of 64.4% from the influent to the effluent. Therefore, at W2, the greatest reduction in MPs (40.7%) was achieved thanks to the coarse and fine grid, aerated grit chamber, and primary settlement tank. In fact, several studies [42,43] showed that most of the MPs could already be removed during the primary treatment phases, mainly through skimming and sedimentation processes, and this high reduction could be due to the fact that many MPs particles were prone to adhere to suspended solids in wastewater. At W3, there was a further reduction in MPs (16%), thanks to the biological treatment with activated sludge, which allows one to remove dissolved organic matter and nutrients through the activity of microorganisms. Finally, a further reduction of MPs (7%) was achieved at W4 during the chlorination disinfection process. The authors report that plastic degradation could be due to long-term contact with chlorinated water, as MPs particles can react with chlorine to produce an oxidative reaction on MPs, although the level of degradation may not only depend on the contact time, but also the temperature and the concentration of chloride in the solution. Hence, it is possible to hypothesize that smaller sized MPs particles may be degraded or broken down below the microscope detection line. However, the authors in their study did not provide convincing conclusions in this regard.

As regards to the average size of the MPs detected, a decreasing trend was found from W1 (571.5 μm) to W2 (235.7 μm), an increase at W3 (422.1 μm), and a new reduction at W4 (348.2 μm). The reduction from W1 to W2 is attributable to filtration of the coarse and fine grid and absorption of sludge in primary sedimentation. The second reduction from W3 to W4 could be associated with chemical degradation due to chlorination disinfection.

The two MPs particles most present in all four wastewater sampling sites were fibers and fragments, with an average percentage between 33.5–56.7% and 30.4–45.6%, respectively. Overall, these data are in line with other studies [17,42]. Mason et al. [44] reported that the highest fraction of MPs in wastewater was fiber (59%), followed by fragments (33%). Instead, Murphy et al. [17] reported that wastewater contains more fragments (67.3%), followed by fibers (18.5%). These studies have in common that fibers and fragments are the two dominant MPs particles in wastewater, depending on the source and type of wastewater.

After the treatment process, the fiber and fragment fraction did not show a significant difference between influent and effluent (*p* > 0.05). The fragment particles present in the wastewater were between 0.02 and 4.2 mm in size, the average size was 0.2 mm, but 70.5% of the fragments were between 0.02 and 1 mm in size. Furthermore, the average fragment particle size was significantly reduced, from 339.1 μm in W1 to 66.5 μm in W4. The smaller presence of larger fragments could be due to mechanical erosion, brittleness, and fracture, but also to chemical and biological degradation; another eventuality could be that heavy fragment particles with a larger size tend to be adsorbed and settled into the sludge. Thus, the larger particles were dominated by the fibers, while the smaller particles were dominated by the fragments and this is in agreement with the data reported by Mason et al. [44].

After Raman analysis, it was confirmed that 81.4% of the suspect particles were MPs. The most common type was PA (polyammide) (54.8%), followed by PP (polypropylene) (9.6%), PE (polyethylene) (9.0%), and PVC (polyvinylchloride) (2.5%). The remaining portion had some other polymers with lower frequency, e.g., PC (polycarbonate), acrylonitrile butadiene styrene, and polyvinyl acetate. PA (nylon) could be released from the washing waters of various types of fabrics, clothes, and carpets. PE and PP may also come from fibrous material (e.g., carpet, clothing). However, the authors found that the PE particles had the same shape and size range as the microspheres found in body care products such as face scrubs and toothpaste. Indeed, the WWTP of this study is located in an area of urban-suburban integration and therefore, it is possible to find plastic particles such as PE that are part of the personal care products. At the end of the process, the WWTP showed a total removal efficiency of 64.4% and also in this case, despite a good removal of the MPs, the main problem is due to a consistent release of microplastics from the WWTP effluent towards the aquatic environment.

Liu et al. [31] evaluated the MPs removal efficiency of a WWTP located in Hvidovre, Denmark. The sampling was carried out in June 2018, taking samples from the four levels of the biofilter and at the outlet of a plastic-free volumetric pump.

The MPs were classified by size, mass, and polymeric composition of the particles retained by the filter and their identification was carried out by FTIR spectroscopy. Among the polymers identified, acrylic had the largest average size (80.3 μm) and PU (polyurethane) the smallest (29.1 μm).

The highest concentration of MPs was detected at the inlet (24.8 μg m^−3^ and 916.8 item m^−3^), while the lowest was at the second sampling level (1.5 μg m^−3^ and 111.1 item m^−3^). The maximum removal efficiency was at level 2 in terms of particle number (87.9%) and at level 4 in terms of particle mass (95.6%). The total removal efficiency was 78.5% for the number of particles and 88.9% for the mass of the particles.

Polyethylene (PE) was the most abundant polymer in terms of particle mass (38%), followed by PP (polypropylene), PVC (polyvinyl chloride), polyester, PS (polystyrene), acrylic, and PA (polyamide). Regarding the number of particles, polyester (34%) was the most abundant polymer, followed by PE, PP, PVC, PS, PA, and acrylic. Epoxy and PU (polyurethane) were rare and less than 1%, both in mass and in number of particles. The high presence of PE, PP, and polyester has also been reported in other treated waters and released by effluents from other WWTPs [32,43,45], thus acting as a carrier for the transport of the MPs not removed from the WWTP in the environment.

Maximum removal of plastic particles was achieved in the upper part of the biofilter, while in the deeper layers, there was limited additional treatment efficiency. The biofilter was able to retain the large MPs particles and no particles larger than 100 μm were found in the final effluent. Although the biofilter reduced the abundance of MPs in the treated wastewater, complete removal was not achieved, as we have seen in the other studies included in this review, thus representing a concrete risk to the environment.

Magni et al. [32] investigated the treatment of MPs in one of the largest WWTPs in Northern Italy which receives water from combined sewers.

The wastewater was sampled for three days of a week and in three different stages of treatment: inlet (IN), after settler (SET), and outbound (OUT). The plastic particles were classified by size, mass, and polymeric composition, carrying out their identification using μFTIR.

The authors distinguished MPPs (microplastic particles) and MPFs (microplastic fibers) on the basis of the different origin and abundance. A MPPs value of 2.0 ± 0.3 MPPs/L was found in the wastewater in the inlet, which was reduced to 0.6 ± 0.2 MPPs/L after the settler, and 0.3 ± 0.1 MPPs/L in the outlet; MPFs had a value of 0.5 ± 0.1 MPFs/L in the inlet, 0.3 ± 0.2 MPFs/L after the settler, and 0.10 ± 0.03 MPFs/L in the outlet, for a total amount of detected MPs of 2.5 ± 0.3 MPs/L in inlet, 0.9 ± 0.3 MPs/L after the settler, and 0.4 ± 0.1 MPs/L in the outlet.

To evaluate the significant differences (*p* < 0.05; *p* < 0.01) about MPs content between the three different treatment steps (IN, SET, and OUT), the authors performed one-way ANOVA; each difference, treatment versus treatment, was evaluated using the Fisher LSD post hoc test. The authors report a significant effect of the treatment steps on the MPs content in wastewater (F_2.6_ = 50.3; *p* < 0.01), with a significant difference in MPs concentration between inlet and settler (*p* < 0.01) and inlet and outlet (*p* < 0.01); no significant difference was observed between settler and outlet (*p* = 0.07).

The WWTP of this study receives on average 400,000,000 L of wastewater/day, treating approximately 1,000,000,000 MPs, with a daily release in surface waters of approximately 160,000,000 MPs. The main form of MPPs in the influent were films (73%), followed by fragments (21%) and lines (6%). After the settler, the percentages of MPPs were films (36%), fragments (36%), and lines (28%), while at the effluent level, the percentages were lines (41%), films (38%), and fragments (21%). The predominant size of MPPs in the collected samples was 0.5–0.1 mm, representing 36% of the total particles in the inlet, 58% after the settler, and 52% in the outlet. A microplastics removal efficiency of 94% was observed for the MPs range size of 5–1 mm and 1–0.5 mm, 77% for the MPs size range of 0.5–0.1 mm, and 65% for MPs size range of 0.1–0.01 mm.

The main classes of MPs in the inlet were acrylonitrile-butadiene copolymer (40%), followed by polyethylene (17%) and ethylene-propylene (14%). After the settler, a high concentration of polyesters (23%), polyethylene (13%), polyurethane (13%), polyamide (11%), and polypropylene (11%) was observed, while in the outlet, the main polymers were polyesters (35%), polyamide (17%), and polyethylene (10%). As for MPFs, in addition to natural fibers (66%), the main class was represented by polyesters with 83% in the inlet, 79% after the settler, and 89% in the outlets; the other polymers were polyacrylates (12%, 8%, and 11% in the three steps) and polyamide (5% in the inlet and 13% in the settler). The MPs removal rate between inlet and outlet was 84% and the largest removal (64%) occurred between inlet and settler.

The authors highlight that MPs have an average value in the influent of 2.5 ± 0.3 MPs/L, a value that is much lower when compared with other influent WWTPs present in Europe, such as those of the UK (15.7 ± 5.2 MPs/L) and Finland (57.6 ± 12.4 MPs/L) [17,43]. These notable differences may be due to several factors. In fact, MPs found in civil wastewater mainly derives from personal care products (PCPs) and synthetic fabrics [7], therefore different individual habits, but also meteorological and seasonal conditions, can contribute to their presence in WWTPs influents from different countries. Another important factor, especially in Italy, is the infiltration of groundwater entering the sewerage system, diluting the concentration of MPs in the wastewater and moreover, in Italy about 70% of the national sewerage system consists of mixed sewers that collect domestic wastes with rainwater runoff and industrial wastewaters all in the same pipe. The different concentrations of MPs detected can also be related to the use of different methods of detection of MPs and for this reason, it is essential to establish common standardized protocols for monitoring MPs, as well as a uniform unit to express the abundance of MPs, so as to simplify the comparison of data between different sampling sites.

Mintenig et al. [33] investigated the presence of MPs in the effluents of 12 WWTPs (Brake, Varel, Oldenburg bp (before post filtration), Oldenburg ap (after post filtration), Berne, Essen, Scharrel, Lohne, Holdorf, Neuharlinigersiel, Schillig, Sandstedt, Burhave) in Lower Saxony, Germany. The treated water comes from municipal and industrial effluents. Six WWTPs have primary skimming tanks where the lighter floating materials are removed from the water surface and then further treated as primary sludge. In all WWTPs, a secondary treatment takes place by reducing organic materials and nitrogen and phosphate compounds and in addition, a tertiary treatment is carried out in four plants to try to further reduce the suspended material.

The collection of water samples coming from the effluents of the 12 WWTPs was carried out between 22 and 29 April 2014.

After sampling, the samples were treated with a multi-step, plastic preserving enzymatic maceration and density separation using a zinc chloride (ZnCl_2_) solution in order to remove the natural organic and inorganic material, respectively, before carrying out the analysis in FITR spectroscopy. This analysis allows one to identify the polymers up to a size of 20 μm. The enzymatic-oxidative purification is an alternative procedure used to prevent the risk of partial or total loss of some polymers, unlike some aggressive chemicals that are usually used for the removal of organic matter. While it has this advantage, enzymatic purification requires a considerable amount of time and effort. In addition, the risk of contamination or sample loss increases due to the different filtration steps that are required for the sample incubation at an optimal pH. The use of zinc chloride solution was preferred over the safer and cheaper sodium chloride solution because it ensured the separation of all common polymers.

In all the effluents examined, MPs > 500 μm with a range between 0–5 × 10^1^ m^−3^ and MPs < 500 μm with a range between 1 × 10^1^ and 9 × 10^3^ m^−3^ were found. No correlations between MPs numbers, sizes, or polymers of respective WWTPs were found.

MPs > 500 μm were detected in 10 of the 12 WWTPs effluents. Only the effluents in Schillig and Oldenburg (ap) did not contain MP > 500 μm. The plastic particles most present among the MPs > 500 μm were polyethylene (PE), with an average of 59%, followed by PP (polypropylene) (average 16%). For each WWTP, the annual discharges of MP > 500 μm were calculated and showed a range from 1 × 10^6^ y^−1^ in Lohne to 5 × 10^7^ y^−1^ in Varel.

MP < 500 μm were contained in all samples, including Oldenburg (ap) and Schillig. In MPs < 500 μm, PE (polyethylene) was always the one with the highest average (40%), followed by polyvinyl alcohol (PVAL), with an average of 16%, and finally, PA (polyamide) and PS (polystyrene) (each 8%). In 59% of the samples examined, the plastic particles were between 50 and 100 μm, while only 4% were larger than 250 μm. The MPs < 500 μm varied considerably between the effluents of Neuharlingersiel (8 × 10^1^ m^−3^), Essen (7 × 10^2^ m^−3^), and Holdorf (9 × 10^3^ m^−3^). In Oldenburg, the post-filtration decreased the amount of MP < 500 μm from 2 × 10^2^ m^−3^ to 1 × 10^1^ m^−3^.

Also, the annual discharges of MP < 500 μm were calculated and showed a range from 1 × 10^7^ y^−1^ in Neuharlingersiel to 5 × 10^9^ y^−1^ in Holdorf.

FITR analysis were conducted for fibers with a size >500 μm and a total of 2 × 10^4^ fibers were detected. Three different polymers (PA, PP, and PEST) were contained in all samples collected. The predominant polymer was PEST (av. 74%), followed by PA (av. 17%) and PP (av. 9%). Discharges of synthetic fibres ranged from 1 × 10^2^ m^−3^ in Burhave to 5 × 10^3^ m^−3^ in Holdorf. In Oldenburg, after post filtration, the load of synthetic fibers was reduced from 9 × 10^2^ m^−3^ to 2 × 10^1^ m^−3^. The annual discharges of fibers were calculated for each WWTP and showed a range from 3 × 10^7^ y^−1^ in Burhave to 3 × 10^9^ y^−1^ in Holdorf.

The use of tertiary treatments, as reported by Akarsu et al., [27] had a low impact for the removal of MPs, however a high removal rate (97%) was detected in the Oldenburg (ap).

Overall, the WWTPs included in this study achieved an MPs removal efficacy of 96% at the end of the treatment processes.

Murphy et al. [17] examined the effectiveness of a secondary WWTP located on the River Clyde, Glasgow (Scotland) for the removal of MPs from municipal effluents.

Sampling was done for all four stages of the treatment process: influent (S1), grit and grease effluent (S2), primary effluent (S3), and the final effluent (S4) before being released into the River Clyde.

MPs were classified according to color, length, and type (fiber, bead, flake, etc.) and identification was performed through FTIR spectroscopy.

430 plastic items (*n* = 303 from the liquid fraction, *n* = 79 from the solid fraction, *n* = 48 from the 24 h sludge cake duplicate) were identified in all samples tested and of these, eight were >5 mm. A significant difference in the amount of MPs was found between the four sampling sites after one-way ANOVA (*p* = 0.0002). At the level of S1, there were on average 15.70 ± 5.20 MP L^−1^, which decreased by 98.4% at the level of S4 (0.25 ± 0.04 MP L^−1^). Although this WWTP showed a remarkable effectiveness in removing MPs, from an estimate on data from the last three years, the authors report that about 65,000,000 MPs can be released daily into the final effluent for a total of about 23 billion MPs per year.

The greatest reduction in the quantity of microplastics (44.59%) was achieved at the S2 level (removal of sand and grease); this was further reduced by 33.75% by the primary settlement tanks and finally, aeration and clarification reduced the amount by 20.07% prior to release from the effluent.

A significant negative correlation was found between the treatment phases and the number of MPs L^−1^ (*p* = 0.014). The most commonly found polymers in S1 were: alkyds (28.7%), polystyrene-acrylic (19.1%), polyester (10.8%), polyurethane (8.9%), and acrylic (8.3%); while those in S4 were: polyester (28%), polyamide (20%), polypropylene (12%), acrylic (12%), alkyd (8%), polyethylene (4%), polystyrene (4%), and PET (4%). In the liquid fraction, we mainly found: flakes (67.3%), fibers (18.5%), film (9.9%), beads (3.0%), and foam (1.3%).

Data analysis showed a statistically significant difference as to the size (mm) of the MPs between the liquid fraction, solid fraction, and 24 h SC duplicate study (*p* = 0.002).

In the liquid fraction, the MPs were an average size of 0.598 mm (±0.089) and were significantly smaller than both the solid fraction 1.342 mm (±0.519) and the 24 h SC duplicate study 1.618 mm (±0.394) (*p* = 0.002).

In the liquid fraction, 78.34% of the MPs were removed through preliminary and primary treatment. Preliminary treatment allows the removal of large objects such as rags and sticks, floating objects, but also sand and grease that can damage or interfere with the treatment system. The primary treatment, through the use of chemical additives (flocculants) and sedimentation, allows the removal of a portion of the suspended solids and organic matter. A further 20.1% of MPs was removed by secondary treatment, which involves the removal of biodegradable organic matter and suspended solids during the aeration and clarification treatment.

Park et al. [34] performed a study to detect the presence of MPs in influents and effluents of 50 municipal WWTPs in South Korea. The authors evaluated the characteristics of the microplastics present in WWTPs, taking into account the type of material, morphology, size, and removal efficiency. The effluents from 14 plants discharge into coastal waters, while the others discharge into rivers and lakes. Further, 39 (78%) of the 50 WWTPs apply anaerobic-anoxic-aerobic (A2O) or modified A2O treatment processes.

The sampling was carried out between 5 September and 1 November 2018, excluding rainy days. The confirmation and identification of the material type was performed with FTIR spectroscopy and the identified microplastics were divided into fibers and fragments.

A total of 1720 microplastic particles were identified in the 50 WWTPs, with 700 and 1020 particles found in the tributaries and effluents, respectively. The concentration of MPs ranged from 10 to 470 MPs/L^−1^ in influents and from 0.004 to 0.59 MPs/L^−1^ in effluents. The size range of the microplastics recovered in this study was 45 μm to 5 mm.

The dominant typology, both in influent and in effluent, were the fragments, and precisely, in influents, 68.2% were fragments and 31.8% were fibers, while in effluents, 82.3% were fragments and 17.7% were fibers. The percentage of fragments found in the effluent was higher than those found in the influent and this could be attributed to sampling differences between the influents and the effluents. In fact, fibers can be washed off the sieve more easily than the fragments, and moreover most of the isolated fibers had smaller diameters than the pore size of the stainless filter used (45 μm). A further explanation is that fibers, unlike microplastic fragments, have an elongated shape, which makes them subject to entanglement during wastewater treatment and gravitational sedimentation.

The material most present in both influents and effluents was PP (polypropylene). In influent samples, PP was present for 39.6%, followed by PE (polyethylene) (25.6%) and PET (polyethylene terephthalate) (21.3%). In addition, other materials such as polystyrene, acrylics, polyamide, polyurethane, and polyether were also present. In the effluent samples, PP was present for 63.3%, followed by PE (13.8%) and PET (13.3%).

Both in the influents and in the effluents PP, PE, and PET were the most present materials, but the amount of PP was much greater in the effluents than in the influents. The predominance of PP in the study by Park et al. [34] is in line with a study by Long et al. [6], which investigated Chinese WWTPs, while Kang et al. [46] report that among the various WWTPs, there are large variations in the amount of polyethylene (15–78%). The high presence of PP could be due to the production, export, and use of many consumer products in plastic material and which are therefore found in the WWTPs of South Korea.

The MPs removal efficiency was calculated only for 31 WWTPs and this is due to the differences in the cutoff size and sampling method employed between the influents and the effluents, obtaining values between 98.7–99.9%, but it is not clear if even microplastics smaller than 100 μm are removed with the same efficiency as larger microplastics. The authors highlight that 16 of the 31 WWTPs were equipped with advanced phosphorus removal units, so they showed greater removal efficiency (Student’s *t*-test, *p* = 0.047) than the other 15 plants that were not equipped with such units. However, no statistically significant differences were identified among different phosphorus removal unit processes. Despite the high efficacy of MPs removal showed by the 31 WWTPs, the lack of data for the other 19 plants represents a fundamental point to have a complete picture of the situation in South Korea regarding the treatment of MPs.

Wang et al. [35] reported the presence, composition, and distribution of MPs in the influent and effluent of several WWTPs in Wujin District, Changzhou (China).

Secondary treatment was applied in all WWTPs. The sampling was carried out in March 2019 by collecting the waters of the influents and effluents of nine domestic WWTPs (W1-W9); in addition, sampling was also carried out for the effluents of five industrial WWTPs (IW1-IW5), for the wastewater of 10 industrial plants (I1-I10), for four livestock farms (A1-A4), and for four fish ponds (F1-F4). The samples collected were pretreated with specific enzymes and H_2_O_2_ to digest organic compounds, while inorganic compounds were removed through density separation. The quantification of MPs was expressed as the number of MPs per liter of water (n L^−1^). The MPs collected were classified according to their size (<100 μm, 100–200 μm, 200–500 μm, 500–1000 μm, 1000–2000 μm, 2000–5000 μm based on the length), shape (fragments, films, pellets, and fibers) and color and were characterized using Raman spectroscopy.

In the influents and effluents of domestic WWTPs, the MPs found were 18–890 and 6–26 n L^−1^, respectively, with a removal efficiency between 35% and 98%. In the effluents of industrial WWTPs, the MPs were 6–12 n L^−1^ and the levels of MPs in the wastewater of industrial plants, livestock farms, and fishing tanks were 8–23, 8–40, and 13–27 n L^−1^, respectively.

Among the MPs found in the collected samples, polyethylene (PE), polypropylene (PP), and polystyrene (PS) made up almost 83% of the total. The most abundant forms were fragments and films and most MPs were <500 μm in size.

In influents of domestic WWTPs, six types of polymers were identified: polyethylene (PE), polypropylene (PP), polystyrene (PS), polyvinyl chloride (PVC), polyethylene terephthalate (PET), and polyamide (PA). Compared to influents, the abundance of MPs in the effluents decreased significantly (Kruskal-Wallis ANOVA, *p* < 0.01), indicating that MPs can be efficiently removed by WWTPs. All WWTPs applied secondary treatment and achieved a removal efficiency that varied considerably between the different WWTPs (35–98%), with an average value of 56%. Furthermore, no significant differences in MPs removal efficiency were found among different secondary treatment technology (*p* > 0.05), demonstrating that removal efficiency can be influenced by treatment technology and operating conditions.

A statistically significant difference (*p* > 0.05) was not observed between the effluents of industrial purification plants and the waste water of industrial plants as regards to the abundance and characteristics of MPs. The industries, therefore, constitute sources of contamination from MPs and in the city of Changzhou, the influents of industrial wastewater mainly come from textile, machine manufacturing, chemical, and steel industries.

Plastic particles were identified in the wastewater of four livestock farms, specifically PE (38%), PA (32%), PS (14%), PP (8%), and PET (8%), and all wastewater collected from fish ponds contained MPs. No significant differences were found in MPs abundance between effluents or wastewater from domestic, industrial, agricultural, and aquaculture sources, indicating that they were all potential sources of MPs pollution. Also, the type and shape of the polymer present in the different sources did not show a statistically significant difference, but differences were found regarding the size and color of the MPs detected in the different sources.

Xu et al. [36] collected and analyzed water samples from the influent and effluent of the major 11 WWTPs in Changzhou, China, with the aim to evaluate the effectiveness of plant treatment and at the same time, to evaluate the abundance, size, color, and shape of MPs in influents and effluents. The 52% of the wastewater treated in these WWTPs came from domestic wastewater, while the remaining 48% came from industrial wastewater.

The sampling was carried out in July 2018, in 2 days, both for the influent and effluent of the 11 WWTPs. The MPs present in each sample were counted and classified according to their shape and were characterized using the FTIR spectroscopy.

In the water samples of the influents of the 11 WWTPs, the MPs ranged from 79.33 ± 0.94 n/L to 342.67 ± 7.37 n/L, with a mean of 196.00 ± 11.89 n/L; in the effluent, the abundance of MPs was between 3.63 ± 0.46 n/L and 13.63 ± 2.63 n/L, with a mean of 9.04 ± 1.12 n/L.

Based on the size, the abundance of MPs present in the influents of the 11 WWTPs was: 0.1–0.5 mm > 0.5–1 mm > 1–5 mm > 0–0.1 mm; also, in the effluents, there was the same order. The size between 0.1 and 0.5 mm of the MPs particles in the influent and effluent was the most present with 51.46% and 41.77%, respectively.

The MPs found in the influents and effluents have been divided according to their shape into fibers, spheres, flakes, films, and fragments and the largest share was represented by fibers, both in the influents (88.69%) and in the effluents (86.66%). MPs fibers are released into the environment mainly from clothing, fabrics, and industry, while flake, film, and fragments mainly derived from the decomposition of plastic, the production of industrial raw materials, and packaging.

Fourteen types of polymers were found, among which those most present in the influent were: 41.79% rayon, 27.60% polyethylene terephthalate (PET), 15.52% polypropylene (PP), 6.10% polyethylene (PE), 3.35% polystyrene (PS), and 2.08% polypropylene copolymer (PE-PP); while in the effluent, they were 43.45%, 29.22%, 14.46%, 6.28%, 2.12%, and 1.51%, respectively.

The MPs removal effectiveness of the 11 WWTPs was almost over 90% and could be as high as 97.15% in WWTP number 4; despite all, a significant amount of MPs are released into the aquatic environment. Due to the inconsistency of the methods of collection, separation, and identification of MPs, as well as the diversification of the quality and quantity of wastewater, the characteristics of pollution caused by MPs released by WWTPs are very different and not always easy to identify.

Yang et al. [37] evaluated the MPs efficiency removal from municipal wastewater of a treatment plant located in Chaoyang district, Beijing (China).

This plant treats mostly domestic effluents and the treatment processes include an aerated grit chamber, primary sedimentation tank, secondary sedimentation tank following A2O treatments (anaerobic, anoxic, and aerobic), and a series of treatment processes like denitrification, ultra- filtration, ozonation, and ultraviolet.

Sampling was carried out from April to June 2018 and in total, 18 types of polymers were detected through μFITR spectroscopy and only two polymers, PET, and phenol-Formaldehyde resin (PF) were detected in all samplings. Polymers detected decreased from influents (6–10 types of polymers) to effluents (4–6 types of polymers), showing that most of the microplastics were effectively removed. Furthermore, no noticeable difference for the polymer composition was found between the samples.

Overall, the most prevalent microplastics were PET (42.25%), polyester (PES) (19.09%), and polypropylene (PP) (13.05%) and they accounted for greater than 70% of the total microplastics. PET and PES were mainly detected as fibers that could be released from household effluents. The fraction of PE showed values (1.64%) rather lower than those reported by other studies conducted in Europe (average 14%) [39] and in the United States (>90%) [42], highlighting potential differences in the use of microplastics between China and Europe/United States.

Microplastics were further classified into microfibers or microparticles. Microfibers (85.92% of microplastics) had an average size of 1110.72 ± 862.95 μm, while microparticles, which included spherical, granule, fragment, film, and irregular shapes, accounted for 14.08% of microplastics and had a mean size of 681.46 ± 528.73 μm, with most of the particles having a size of about 300 μm. The method used did not allow the extraction and quantification of microparticles with dimensions <50 μm.

In influent, 12.03 ± 1.29 items/L were detected, but after the treatments (primary aerated grit, A2O, and a series of advanced treatment), there was a statistically significant reduction (post hoc Tukey test, *p* < 0.05) and in fact, the authors counted 0.59 ± 0.22 items/L of microplastics in the final effluent, for a total reduction of 95%. This result was similar to other effluents located in Scotland (0.25 ± 0.04 items/L) [17] or in Australia (0.28 items/L) [47], but was slightly higher than the effluents in Europe [39] and the USA [44].

**Figure 2:**
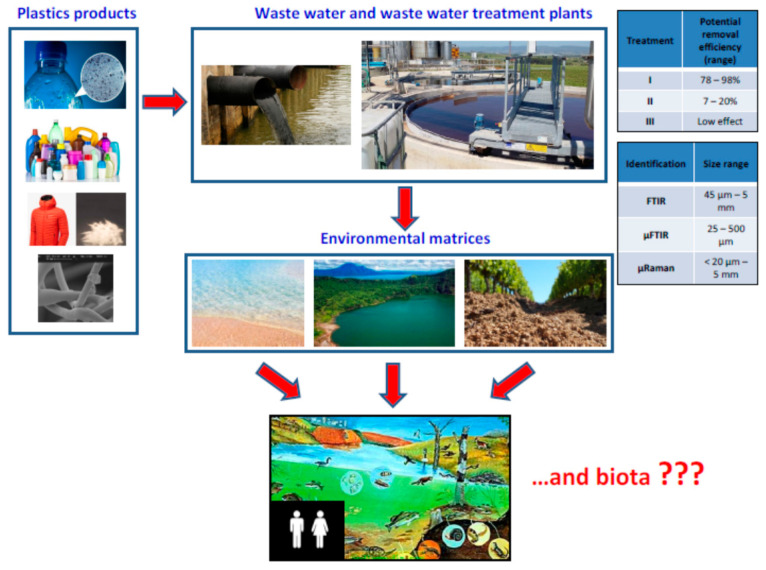
Release of microplastics (MPs) from everyday products in water destined for wastewater treatment plants (WWTPs) and their environmental fate.

The 15 selected full research articles allowed us to have an overview of the main types of WWTPs and their efficiency in the process of removing MPs from wastewater. In the selected studies of this systematic review, we have observed how WWTPs located in different continents (Europe, Asia, North America) mostly use a primary and secondary type of treatment that allows one to reach a high percentage of MPs removal from wastewater (Figure 2). Briefly, the primary treatment is a physical process used for the removal of part of the sedimentable substances contained in the wastewater, which may include screening, sand removal, degreasing, and primary sedimentation; secondary treatment is usually a biological process used for the removal of sedimentable and non-sedimentable organic substances contained in the wastewater and includes an aerobic phase, through the insufflation of air, and secondary sedimentation or filtering for the separation of excess sludge; the tertiary treatment is carried out on the effluent leaving the secondary treatment and it should enable the breakdown of those substances that are not eliminated during the primary and secondary treatments. Akarsu et al. [27] and Magni et al. [32] confirmed that tertiary treatment does not significantly contribute to the increase in the removal efficiency of MPs (Figure 2), however Mintenig et al. [33] reported that in the Oldenburg (ap) plant, the tertiary treatment had contributed (coupled with primary and secondary) to achieving a high removal efficiency (97%).

Despite the high removal efficiency of WWTPs, the main problem continues to be represented by the release of MPs from WWTP effluents into the aquatic environment (Figure 2), as well as from the extraction sludge in the soil, as they are often used in agriculture as fertilizers. However, if we consider that wastewater treatment plants were not designed exclusively for the removal of microplastics, the efficiency rates achieved by most WWTPs included in this study are quite high. Another problem is that the microplastics present in the samples from wastewater are difficult to identify because they are rich in organic and inorganic substances and then, several technologies have been developed to separate microplastics from impurities like pretreatment, oxidation, enzymatic digestion, flotation, sedimentation, etc. [2].

The characterization of MPs is also of fundamental importance, which is divided into physics and chemistry. Physical identification is done by visual examination with a microscope and plastic microparticles are classified according to size, shape (fiber, film, foam, pellet, or fragments), and colour [1]. In order to discriminate between plastic and non-plastic, several authors [1,48,49] reported that the microparticles found must have precise characteristics: the samples cannot have organic matter, the fibers must have the same thickness along their entire length, and the particles must show a homogeneous colour along their structure. However, visual examination is not sufficient for the identification of MPs because as the particle size decreases, the number of errors increases and therefore, it is also necessary to carry out the assessment of the chemical structure. Visual identification can also be performed by scanning electron microscopy (SEM), a method that allows one to study the morphology of particles and thanks to the high resolution of the images, allows the identification of impurities and possible MPs. The assessment of the chemical structure is important because it allows us to identify the various types of MPs and also provides the polymer base and even the presence of additives, giving us further information regarding society habits and waste disposal. In the selected full research articles, the analytical methods used were FTIR or Raman spectroscopy, which are also the most commonly used techniques (Figure 2).

However, the reading conditions in these techniques depend on the characteristics of the sample since size, shape, and colour can interfere with the analysis and therefore, it is necessary to make adjustments in the equipment in order to obtain an adequate spectrum with less noise and clearly evident peaks. For the identification of larger particles (>500 μm), ATR-FTIR has proved to be an efficient type of analysis. In identifying MPs by FTIR, it is possible to measure transmittance and reflectance, including the configuration of the total attenuated reflectance (ATR). ATR-FTIR coupled with a microscope (μ-ATR-FTIR) is used to analyze smaller particles.

Raman analysis shows a higher sensitivity that allows one to identify even small particles (<20 μm), but it may be subject to interference by the additives present in commercial plastics, thus causing changes in the spectrum of the base polymer which makes identification difficult. Taking this into account, Käppler et al. [50] suggest using both FTIR and Raman to obtain more complete and accurate results of the analyzed particles [1].

Most of the WWTPs included in this review receive municipal and/or industrial wastewater and the predominant forms detected in effluents were fibers and fragments, with percentages often higher than 60% and 40%, respectively; only Magni et al. [32] reported the predominance of films (38%) over fragments (21%). The type of polymer most present was PE in almost all WWTPs investigated in this review, with the exception of the studies by Liu et al., [30] where the predominant polymer was PA (54.8%). For Magni et al., [32] it was polyester (35%); for Park et al., [34] it was the PP (63.3%); for Xu et al., [36] it was rayon (41.79%); and for Yang et al., [37] it was PET (42.25%). PE mainly comes from fibrous material (clothing, fabrics, carpets, etc.), however this polymer has been widely used in body care and personal hygiene products, demonstrating that this polymer is not only found in WWTPs that receive industrial effluents, but also those that receive municipal waters. PA is often released from the water used for washing various types of fabrics, clothes, and carpets, as well as rayon, PES, and PET as these polymers are also used in the production of synthetic textile fibers and their release takes place through domestic and/or industrial discharge. PP mainly comes from the production, export, and use of many plastic consumer products, so its release is linked in particular to industrial and commercial activities. In fact, the greatest percentages of this polymer were found in the industrial effluents of WWTPs, in particular, in the Korean and Chinese ones, as reported by Park et al. [34].

The size of the plastic particles is also a relevant concern because small particles are difficult to remove during treatment and therefore, they can be released into the environment. This phenomenon can be influenced by the possible fragmentation processes that occur during the treatments and therefore, not only their removal becomes more difficult, but also their identification. Our review shows the MPs with the smallest size were found in the studies by Liu et al. [30] and Liu et al. [31] with a size of 20 and 29.1 μm, respectively.

Once the microplastics are released into the environment, they will interact with living organisms and the possible effects on human health still need to be verified (Figure 2).

Although there is no reliable data yet, it appears that microplastics can induce oxidative stress in cells due to their chemical composition and the pollutants that they can carry across their surface, which in turn cause oxidative stress in cells. How microplastics can pose an ecotoxicological threat is still a controversial topic that requires further study and several groups have conducted acute and chronic toxicity tests on different aquatic invertebrates [5]. Human health could be threatened by microplastics because they are known to accumulate in some species of fish and crustaceans (wild or aquaculture), but also in vegetables and fruit, and in particular, their toxicity could be due to additives or adsorbed chemicals. There is still not sufficient information to assess human exposure to microplastics through food and there is also a lack of toxicological and epidemiological data [5]. However, Oliveri Conti et al. [51] reported a study aimed at evaluating dietary exposure to MPs that may be contained in the most commonly consumed fruits and vegetables, also in relation to their recommended daily intake. MPs extraction and analysis were carried out using an innovative Italian methodology and SEM-EDX, respectively, which made it possible to identify plastic particles significantly smaller than 10 μm (size 1.51 μm).

It is essential to reduce the release of these particles into the environment and in this regard, it could be useful to introduce changes not only in purification plants, but also in washing machines or other domestic appliances so as to reduce the release of the domestic, synthetic, or modified anthropogenic microfibers [2]. In addition, several countries (Canada, Ireland, UK, United States) are working to reduce or ban the use of microspheres in body care products. The production of fabrics can also be improved in order to reduce the release of microfibers [7]. Finally, an alternative solution could be the production of biodegradable plastics, i.e., synthetic polymers that can be decomposed by the action of microorganisms in the environment, producing CO_2_ and H_2_O [2].

## 4. Conclusions

As reported by the studies selected in this review, the removal efficiency achieved by WWTPs was high, despite WWTPs not being designed solely for the removal of microplastics. As a result, millions of MPs continue to be released from these plants every day. Fibers and fragments are the two dominant types of plastic particles in wastewater. Although in some WWTPs it has been possible to remove and identify particles of size equal to 20 μm, further fragmentation of the particles can be obtained following natural processes or during treatment and this will make their removal difficult. For the identification of MPs > 500 μm, ATR-FTIR has proved to be an efficient type of analysis, while μFTIR and μRaman are more suited for smaller particles identification. The type of polymer most present was PE in almost all WWTPs investigated in this review. Small particles are difficult to remove during treatment and therefore, they can be released into the environment. Furthermore, the lowest MPs count were those of 20 μm, which are difficult to identify and measure, so we suggest knowledge should be improved about total MPs content in treated wastewater because this aspect has not yet been evaluated. In fact, nano and microplastics below 20 μm are the particles that have the ability to be absorbed by living organisms through direct and indirect contact (food chain).

To date, no standard protocol or international rules are yet available to be followed by professionals for monitoring of the MPs in treated wastewater and therefore, the methodological differences, especially in the initial stages of sampling and in the selection of the size ranges of the MPs to be analyzed, make it difficult to compare results. Therefore, efficient and rapid protocols should be drawn up and will increase the knowledge of sources and strategies to further reduce microparticles contamination of treated wastewater, with the aim of obtaining even less release into the environment.

## Figures and Tables

**Table 1:** Full research article selected.

Study	WWTPs Location	Sampling Date	WWTP Treatment	Range Size Particles	Spectroscopy Analyses	Types of Polymers	Total Removal Efficiency
[27]	Mersin Bay, Turkey	2017	Primary and secondary treatment (Tarsus and Silifke), Tertiary treatment (Karaduvar)	<500 μm, 500–1000 μm, 1000–2000 μm, 2000–3000 μm	FTIR	PE, PP, PS, ACRYLIC FIBER, CELLULOSE ACETATE	58%
[28]	Region of Murcia, Spain	February 2018–July 2019	Primary and secondary treatment	<1 mm, 1–5 mm	FTIR	LDPE, NYL, PV, MUF, HDPE, AC, PP, PS, MCR, EPM, BPL, PEST, PTFE, PIB	76.5%
[8]	Cartagena, Spain	September 2016–April 2018	Primary and secondary treatment	400–600 μm	FTIR	LDPE, HDPE, AC, PP, PEP, PS, BPL, NYL, PUR, PET, MCR, PTFE, MMF, PES, PVI, PIB, RBB	90.3%
[4]	Charleston, South Carolina, USA	June 2016, October 2016, January 2017, April 2017, July 2017	Primary and secondary treatment	60–178 μm, 178–418 μm, >418 μm	FTIR	Not specify	89.4%
[5]	Madrid, Spain	Spring 2019	Primary and secondary treatment	25–104 μm, 104–375 μm, >375 μm <5 mm	μFTIR	PE, PP, PET, CELLOPHANE, ACRYLIC FIBER, PMMA, PCL, PU, PS	93.7%
[29]	Vancouver, Canada	September and October 2016	Primary and secondary treatment	<500 μm, >500 μm	ATR-FTIR	PEST, PA, PS, PP, NYLON	98.3%
[30]	Wuhan, China	Not specified	Primary and secondary treatment	0.02–4 mm	μRaman	PA, PP, PE, PVC, PC, ACRYLONITRILE BUTADIENE STYRENE, POLYVINYL ACETATE	64.4%
[31]	Hvidovre, Denmark	June 2018	Primary and secondary treatment	29.1–80.3 μm	μFTIR	PE, PP, PVC, PS, ACRYLIC, PA, EPOXY, PU	78.5%
[32]	Northern Italy	Not specified (three days of sampling)	Primary, secondary, and tertiary treatment	0.01–0.1 mm, 0.1–0.5 mm, 0.5–1 mm, 1–5 mm	μFTIR	PE, EPM, PEST, PU, PA, PP, PAC, ACRYLONITRILE BUTADIENE COPOLYMER	84%
[33]	Lower Saxony, Germany	22–29 April 2014	Primary, secondary, and tertiary treatment	>500 μm, <500 μm	μFTIR	PEST, PE, PP, PVAL, PA, PS	97%
[17]	Glasgow, Scotland	Not specified	Primary and secondary treatment	0.598–1.618 mm	FTIR	PEST, PU, PA, PP, PE, PET, PS, ALKYDS, ACRYLIC, PSA	98.4%
[34]	South Korea	September 5th November 1st	Primary and secondary treatment	45 μm–5 mm	FTIR	PP, PE, PET, PS, PA, PU, ACRYLIC, POLYETHER	98.7–99.9%
[35]	Wujin District, Changzhou, China	March 2019	Primary and secondary treatment	<100 μm, 100–200 μm, 200–500 μm, 1000–2000 μm, 2000–5000 μm	μRaman	PE, PP, PS, PVC, PET, PA	35–98%
[36]	Changzhou, China	July 2018	Primary and secondary treatment	0.1–0.5 mm, 0.5–1 mm, 1–5 mm	ATR-FTIR	RAYON, PET, PP, PE, PS, PE-PP	90%
[37]	Chaoyang district, Beijing, China	April–June 2018	Primary and secondary treatment	152.7–1973.67 μm	μFTIR	PET, PF, PES, PP, PE	95%

AC: acrylate; BPL: biopolymer; EPM: poly (ethylene:propylene); HDPE: high density polyethylene; LDPE: low density polyethylene; MCR: methacrylate; MMF or MUF: melamine; NYL: nylon; PA: polyamide; PC: polycarbonate; PE: polyethylene; PF: phenol-formaldehyde resin; PP: polypropylene; PS: polystyrene; PU or PUR: polyurethane; PV or PVI: polyvinyl; PCL: polycaprolactone; PEP: polyehtylene propylene); PES or PEST: polyester; PET: polyethylene terephthalate; PIB: polyisobutylene; PVC: polyvinylchloride; PMMA: polymethylmethacrylate; PTFE: teflon; PVAL: polyvinylalcohol; RBB: rubber.

## References

[1] Bretas Alvim C., Mendoza-Roca J.A., Bes-Piá A. (2020). Wastewater treatment plant as microplastics release source–Quantification and identification techniques. J. Environ. Manag..

[2] Sol D., Laca A., Laca A., Díaz M. (2020). Approaching the environmental problem of microplastics: Importance of WWTP treatments. Sci. Total Environ..

[3] Ziajahromi S., Neale P.A., Leusch F.D.L. (2016). Wastewater treatment plant effluent as a source of microplastics: Review of the fate, chemical interactions and potential risks to aquatic organisms. Water Sci. Technol..

[4] Conley K., Clum A., Deepe J., Lane H., Beckingham B. (2019). Wastewater treatment plants as a source of microplastics to an urban estuary: Removal efficiencies and loading per capita over one year. Water Res. X.

[5] Edo C., González-Pleiter M., Leganés F., Fernández-Piñas F., Rosal R. (2020). Fate of microplastics in wastewater treatment plants and their environmental dispersion with effluent and sludge. Environ. Pollut..

[6] Long Z., Pan Z., Wang W., Ren J., Yu X., Lin L., Lin H., Chen H., Jin X. (2019). Microplastic abundance, characteristics, and removal in wastewater treatment plants in a coastal city of China. Water Res..

[7] Prata J.C. (2018). Microplastics in wastewater: State of the knowledge on sources, fate and solutions. Mar. Pollut. Bull..

[8] Bayo J., Olmos S., López-Castellanos J. (2020). Microplastics in an urban wastewater treatment plant: The influence of physicochemical parameters and environmental factors. Chemosphere.

[9] Zuccarello P., Ferrante M., Cristaldi A., Copat C., Grasso A., Sangregorio D., Fiore M., Conti G.O. (2019). Exposure to microplastics (<10 μm) associated to plastic bottles mineral water consumption: The first quantitative study. Water Res..

[10] Zuccarello P., Ferrante M., Cristaldi A., Copat C., Grasso A., Sangregorio D., Fiore M., Conti G.O. (2019). Reply for comment on “Exposure to microplastics (<10 μm) associated to plastic bottles mineral water consumption: The first quantitative study by Zuccarello et al. [Water Research 157 (2019) 365–371]”. Water Res..

[11] He D., Luo Y., Lu S., Liu M., Song Y., Lei L. (2018). Microplastics in soils: Analytical methods, pollution characteristics and ecological risks. TrAC Trends Anal. Chem..

[12] Brennecke D., Duarte B., Paiva F., Caçador I., Canning-Clode J. (2016). Microplastics as vector for heavy metal contamination from the marine environment. Estuar. Coast. Shelf Sci..

[13] Ferrante M., Napoli S., Grasso A., Zuccarello P., Cristaldi A., Copat C. (2019). Systematic review of arsenic in fresh seafood from the Mediterranean Sea and European Atlantic coasts: A health risk assessment. Food Chem. Toxicol..

[14] Gao F., Li J., Sun C., Zhang L., Jiang F., Cao W., Zheng L. (2019). Study on the capability and characteristics of heavy metals enriched on microplastics in marine environment. Mar. Pollut. Bull..

[15] Fiore M., Barone R., Copat C., Grasso A., Cristaldi A., Rizzo R., Ferrante M. (2020). Metal and essential element levels in hair and association with autism severity. J. Trace Elem. Med. Biol..

[16] Prata J.C., da Costa J.P., Duarte A.C., Rocha-Santos T. (2019). Methods for sampling and detection of microplastics in water and sediment: A critical review. TrAC Trends Anal. Chem..

[17] Murphy F., Ewins C., Carbonnier F., Quinn B. (2016). Wastewater Treatment Works (WwTW) as a Source of Microplastics in the Aquatic Environment. Environ. Sci. Technol..

[18] Guerranti C., Martellini T., Perra G., Scopetani C., Cincinelli A. (2019). Microplastics in cosmetics: Environmental issues and needs for global bans. Environ. Toxicol. Pharmacol..

[19] Kalčíková G., Alič B., Skalar T., Bundschuh M., Gotvajn A.Ž. (2017). Wastewater treatment plant effluents as source of cosmetic polyethylene microbeads to freshwater. Chemosphere.

[20] Vieira G.H.A., Nogueira M.B., Gaio E.J., Rosing C.K., Santiago S.L., Rego R.O. (2016). Effect of Whitening Toothpastes on Dentin Abrasion: An In Vitro Study. Oral Health Prev. Dent..

[21] Browne M.A., Crump P., Niven S.J., Teuten E., Tonkin A., Galloway T., Thompson R. (2011). Accumulation of Microplastic on Shorelines Woldwide: Sources and Sinks. Environ. Sci. Technol..

[22] Carney Almroth B.M., Åström L., Roslund S., Petersson H., Johansson M., Persson N.-K. (2018). Quantifying shedding of synthetic fibers from textiles; a source of microplastics released into the environment. Environ. Sci. Pollut. Res..

[23] De Falco F., Gullo M.P., Gentile G., Di Pace E., Cocca M., Gelabert L., Brouta-Agnésa M., Rovira A., Escudero R., Villalba R. (2018). Evaluation of microplastic release caused by textile washing processes of synthetic fabrics. Environ. Pollut..

[24] Talvitie J., Mikola A., Setälä O., Heinonen M., Koistinen A. (2017). How well is microlitter purified from wastewater?—A detailed study on the stepwise removal of microlitter in a tertiary level wastewater treatment plant. Water Res..

[25] Mohapatra D.P., Cledón M., Brar S.K., Surampalli R.Y. (2016). Application of Wastewater and Biosolids in Soil: Occurrence and Fate of Emerging Contaminants. Water Air Soil Pollut..

[26] Moher D., Liberati A., Tetzlaff J., Altman D.G. (2015). Linee guida per il reporting di revisioni sistematiche e meta-analisi: Il PRISMA Statement. Open Access.

[27] Akarsu C., Kumbur H., Gökdağ K., Kıdeyş A.E., Sanchez-Vidal A. (2020). Microplastics composition and load from three wastewater treatment plants discharging into Mersin Bay, north eastern Mediterranean Sea. Mar. Pollut. Bull..

[28] Bayo J., López-Castellanos J., Olmos S. (2020). Membrane bioreactor and rapid sand filtration for the removal of microplastics in an urban wastewater treatment plant. Mar. Pollut. Bull..

[29] Gies E.A., Lenoble J.L., Noël M., Etemadifar A., Bishay F., Hall E.R., Ross P.S. (2018). Retention of microplastics in a major secondary wastewater treatment plant in Vancouver, Canada. Mar. Pollut. Bull..

[30] Liu X., Yuan W., Di M., Li Z., Wang J. (2019). Transfer and fate of microplastics during the conventional activated sludge process in one wastewater treatment plant of China. Chem. Eng. J..

[31] Liu F., Nord N.B., Bester K., Vollertsen J. (2020). Microplastics Removal from Treated Wastewater by a Biofilter. Water.

[32] Magni S., Binelli A., Pittura L., Avio C.G., Della Torre C., Parenti C.C., Gorbi S., Regoli F. (2019). The fate of microplastics in an Italian Wastewater Treatment Plant. Sci. Total Environ..

[33] Mintenig S.M., Int-Veen I., Löder M.G.J., Primpke S., Gerdts G. (2017). Identification of microplastic in effluents of waste water treatment plants using focal plane array-based micro-Fourier-transform infrared imaging. Water Res..

[34] Park H.-J., Oh M.-J., Kim P.-G., Kim G., Jeong D.-H., Ju B.-K., Lee W.-S., Chung H.-M., Kang H.-J., Kwon J.-H. (2020). National Reconnaissance Survey of Microplastics in Municipal Wastewater Treatment Plants in Korea. Environ. Sci. Technol..

[35] Wang F., Wang B., Duan L., Zhang Y., Zhou Y., Sui Q., Xu D., Qu H., Yu G. (2020). Occurrence and distribution of microplastics in domestic, industrial, agricultural and aquacultural wastewater sources: A case study in Changzhou, China. Water Res..

[36] Xu X., Jian Y., Xue Y., Hou Q., Wang L. (2019). Microplastics in the wastewater treatment plants (WWTPs): Occurrence and removal. Chemosphere.

[37] Yang L., Li K., Cui S., Kang Y., An L., Lei K. (2019). Removal of microplastics in municipal sewage from China’s largest water reclamation plant. Water Res..

[38] Habib R., Thiemann T., Kendi R. (2020). Microplastics and Wastewater Treatment Plants—A Review. J. Water Resour. Prot..

[39] Talvitie J., Mikola A., Koistinen A., Setälä O. (2017). Solutions to microplastic pollution–Removal of microplastics from wastewater effluent with advanced wastewater treatment technologies. Water Res..

[40] Dris R., Gasperi J., Saad M., Mirande C., Tassin B. (2016). Synthetic fibers in atmospheric fallout: A source of microplastics in the environment?. Mar. Pollut. Bull..

[41] Michielssen M.R., Michielssen E.R., Ni J., Duhaime M.B. (2016). Fate of microplastics and other small anthropogenic litter (SAL) in wastewater treatment plants depends on unit processes employed. Environ. Sci. Water Res. Technol..

[42] Carr S.A., Liu J., Tesoro A.G. (2016). Transport and fate of microplastic particles in wastewater treatment plants. Water Res..

[43] Lares M., Ncibi M.C., Sillanpää M., Sillanpää M. (2018). Occurrence, identification and removal of microplastic particles and fibers in conventional activated sludge process and advanced MBR technology. Water Res..

[44] Mason S.A., Garneau D., Sutton R., Chu Y., Ehmann K., Barnes J., Fink P., Papazissimos D., Rogers D.L. (2016). Microplastic pollution is widely detected in US municipal wastewater treatment plant effluent. Environ. Pollut..

[45] Lv X., Dong Q., Zuo Z., Liu Y., Huang X., Wu W.-M. (2019). Microplastics in a municipal wastewater treatment plant: Fate, dynamic distribution, removal efficiencies, and control strategies. J. Clean. Prod..

[46] Kang H.-J., Park H.-J., Kwon O.-K., Lee W.-S., Jeong D.-H., Ju B.-K., Kwon J.-H. (2018). Occurrence of microplastics in municipal sewage treatment plants: A review. Environ. Health Toxicol..

[47] Ziajahromi S., Neale P.A., Rintoul L., Leusch F.D.L. (2017). Wastewater treatment plants as a pathway for microplastics: Development of a new approach to sample wastewater-based microplastics. Water Res..

[48] Hidalgo-Ruz V., Gutow L., Thompson R.C., Thiel M. (2012). Microplastics in the marine environment: A review of the methods used for identification and quantification. Environ. Sci. Technol..

[49] Hidayaturrahman H., Lee T.-G. (2019). A study on characteristics of microplastic in wastewater of South Korea: Identification, quantification, and fate of microplastics during treatment process. Mar. Pollut. Bull..

[50] Käppler A., Fischer D., Oberbeckmann S., Schernewski G., Labrenz M., Eichhorn K.-J., Voit B. (2016). Analysis of environmental microplastics by vibrational microspectroscopy: FTIR, Raman or both?. Anal. Bioanal. Chem..

[51] Conti G.O., Ferrante M., Banni M., Favara C., Nicolosi I., Cristaldi A., Fiore M., Zuccarello P., Maria F. (2020). Micro- and nano-plastics in edible fruit and vegetables. The first diet risks assessment for the general population. Environ. Res..

